# THE ROLE OF HEALTH LITERACY ON PROCESS AND OUTCOMES OF A DIABETES SELF-HELP INTERVENTION WITH KOREAN AMERICANS

**DOI:** 10.1093/geroni/igz038.1511

**Published:** 2019-11-08

**Authors:** Miyong T Kim

**Affiliations:** University of Texas at Austin, Austin, Texas, United States

## Abstract

The purpose of this study was to explicate the underlying mechanisms of the role of health literacy (HL) in diabetes management process involving a group of Korean Americans with type 2 diabetes mellitus (DM). We used data from a randomized clinical trial of an HL-focused diabetes self-management intervention (n = 250). A series of path analyses identified the level of self-efficacy and self-care skills as a significant mediator between HL and glucose control (HbA1C) and quality of life for the target population. In addition, education and acculturation were revealed as the most significant correlates of HL for this new immigrant group. Despite inconsistent empirical findings regarding the statistically significant effect of HL on glucose control, this study confirmed the apriority hypothesis that HL indirectly influences health outcome through mediators such self-care skills as self-efficacy. This study highlighted the importance of HL in chronic disease management for people with limited HL.

